# DNA methylation biomarkers for early detection of gastric and colorectal cancers

**DOI:** 10.20892/j.issn.2095-3941.2023.0443

**Published:** 2024-02-05

**Authors:** Xianchun Gao, Hui Liu, Jun Yu, Yongzhan Nie

**Affiliations:** 1 Key Laboratory of Resource Biology and Biotechnology in Western China, Ministry of Education, Faculty of Life Science and Medicine, Northwest University, Xi’an 710069, China; 2 State Key Laboratory of Holistic Integrative Management of Gastrointestinal Cancers and National Clinical Research Center for Digestive Diseases, Xijing Hospital of Digestive Diseases, Fourth Military Medical University, Xi’an 710032, China

Cancer is one of the leading causes of death worldwide. The early diagnosis of cancer remains one of the greatest cancer research challenges. Epigenetic alterations, such as altered DNA methylation, that occur during the early stages of carcinogenesis have been proposed as candidate cancer biomarkers. In recent years detection of small amounts of methylated DNA in samples, including blood and stool, has demonstrated the feasibility of DNA methylation as a molecular cancer biomarker. The translational promise of aberrant DNA methylation includes screening and detecting cancer, evaluating prognosis, assessing treatment efficacy, and detecting minimal residual disease (**Figure 1**). The application of DNA methylation biomarkers for cancer detection has been studied most intensively. Alterations in DNA methylation patterns in the genome have been observed across malignancies and usually occur before other detectable genetic changes^1^. Therefore, biomarker mining for the early diagnosis of cancer based on DNA methylation has emerged as a promising field and has become a focus of research globally. Although hundreds of DNA methylation biomarkers have displayed great potential for early cancer detection, only a few methylation biomarkers have been used in the clinical setting to date. The National Medical Products Administration (NMPA) in China has approved 20 methylation-based commercial kits for cancer diagnosis. More than one-half of these kits are used for colorectal cancer (CRC) diagnosis (11); one kit is used for gastric cancer, three for cervical cancer, two for lung cancer, and the remaining three are used for the diagnosis of gliomas, and liver and bladder cancers. In the US, seven DNA methylation-based assays are available commercially to help clinicians make better treatment decisions in patients with cancer^2^. Two assays can be used to detect CRC and one can be used to detect > 50 types of tumors.

**Figure 1:**
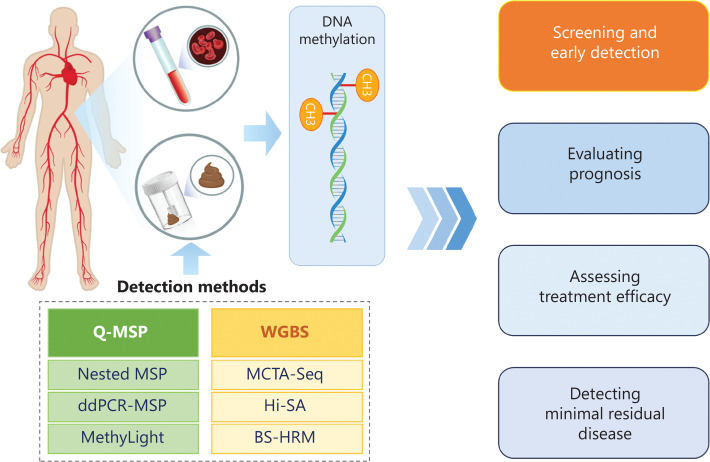
Main technologies for DNA methylation detection and clinical applications.

Unlike Western countries, gastric cancer and CRC are highly prevalent in China with > 480,000 patient-related deaths, accounting for 20.1% of all cancer-related deaths^3^. The incidence of CRC in China has rapidly increased. CRC currently ranks second with respect to morbidity among all malignancies^3^. The incidence of gastric cancer in China is among the highest worldwide, accounting for > 45% of all new gastric cancer cases^3^. Gastric cancer and CRC have a poor prognosis and are difficult to diagnose in the early stages due to a lack of characteristic clinical manifestations. In high-risk groups, endoscopy with tissue biopsies is the gold standard for diagnosing gastric cancer and CRC; however, endoscopy is invasive and highly dependent on the judgment and experience of the endoscopic specialist. Unfortunately, the currently available protein markers, such as CEA, CA19-9 and CA72-4, are ineffective in detecting early-stage gastrointestinal cancer owing to a low sensitivity. There is an ongoing quest for reliable non-invasive biomarkers with better sensitivity and specificity for the detection of gastrointestinal cancer to complement the currently available screening methods. Gastric cancer and CRC share many biological features. For example, both stomach and colorectum epithelia are derived from endoderm. Normal cells undergo a hyperplasia-neoplasia-cancerous process during tumorigenesis to become cancerous. Notably, gastric cancer and CRC share many aberrant DNA methylations, including *SEPT9*, *MGMT*, and *SDC2*. Therefore, in this perspective we focused on the progress in research involving DNA methylation-based diagnostics for gastric cancer and CRC screening and early detection.

## Clinical applications of DNA methylation biomarkers for detecting early gastrointestinal cancers

### Colorectal cancer

Screening for CRC using a fecal immunochemical test (FIT) has been shown to reduce CRC-related mortality; however, a FIT is limited by relatively low specificity and sensitivity for early CRC detection. Recently, several methylated genes have been studied epigenetically as alternative biomarkers to FIT.

#### Blood-based DNA methylation biomarkers for screening and early detection of CRC

To date, several potential blood-based DNA methylation biomarkers have been identified for CRC detection, including *BCAT1, BMP3, C9orf50, CDKN2A, CLIP4, KCNQ5, MLH1, NDRG4, PRIMA1, SDC2, SEPT9, SFRP2*, and *VIM*^2,4^ (**Table 1**). In fact, the best-known blood epigenetic marker for CRC is *SEPT9*. Methylated *SEPT9* is the only single-gene methylation biomarker approved by the U.S. Food & Drug Administration (FDA) for CRC detection, as well as the first methylation biomarker approved by the NMPA in China. Methylation changes in *SEPT9*, a member of the septin family, which is involved in cytokinesis and cytoskeletal organization, have been linked to multiple cancers. In case-control and opportunistic screening studies, plasma methylated *SEPT9* demonstrated approximately 70% sensitivity and 90% specificity for detecting CRC^4^. In a large prospective CRC screening cohort, the sensitivity and specificity of methylated *SEPT9* were estimated to be 48.2% and 91.5%, respectively^5^. Furthermore, among patients with TNM and Duke stage progression, the positive methylated *SEPT9* rates gradually increase^6^. Of note, the criteria for determining methylated *SEPT9* positivity vary across studies. For example, in some studies a positive reaction was indicated by a methylated *SEPT9* curve exceeding the prespecified threshold of 50 polymerase chain reaction (PCR) cycles^5^, whereas a predetermined threshold of 45 PCR cycles was applied in other studies^7^. In addition, there is inconsistency in the PCR repeat systems used across different studies; most studies use triplicate PCR reactions, while other studies use double replicates^5^. Therefore, the methylated *SEPT9* test performance across studies may reflect differences in the study populations, different interpretation thresholds among commercially available kits, and differences between study settings (retrospective case-control study *vs.* opportunistic *vs.* population-based screening).

**Table 1:** Overview of promising DNA methylation biomarkers used in the diagnosis of CRC and adenomas

Gene	Test	Sample type	Cohort size		Sensitivity (%)	Specificity (%)	Method	NMPA approval (date)	Reference PMID
			Controls	Cases					
**Single methylated gene**									
*BMP3*	CRC	Blood	50	45	40	94	BS-HRM	No	29892846
		Stool	40	35	40	85	MSP	No	29142517
	AA	Stool	40	36	33.3	85	MSP	No	29142517
*CDKN2A*	CRC	Blood	10	52	38	100	MSP	No	11801557
		Stool	31	30	40	96.8	MSP	No	21033217
	Adenoma	Stool	31	25	24	96.8	MSP	No	21033217
*MGMT*	CRC	Stool	24	52	48.1	100	MSP	No	17352030
	Adenoma	Stool	24	21	28.6	100	MSP	No	17352030
*MLH1*	CRC	Blood	19	-	33	100	PCR	No	11221878
*NDRG4*	CRC	Blood	16	84	54.8	78.1	Nested MSP	No	25663916
		Stool	16	84	76.2	89.1	Nested MSP	No	25663916
	AA	Stool	40	36	27.8	80	MSP	No	29142517
*PRIMA1*	CRC	Blood	37	47	80.9	73	MSP	No	28753106
	Adenoma	Blood	37	37	70.3	73	MSP	No	28753106
*SDC2*	CRC	Blood	125	131	87.0	95.2	MSP	No	23747112
		Stool	713	359	83.8	98.0	MSP	Yes	33126908
	Adenoma	Blood	37	37	81.1	97.3	MSP	No	28753106
	AA	Stool	713	38	42.1	98.0	MSP	Yes	33126908
*SEPT9*	CRC	Blood	295	291	76.6	95.9	MSP	Yes (2015)	27133379
		Stool	76	72	83.3	92.1	qMSP	No	32373158
	Adenoma	Blood	295	214	9.8	95.9	MSP	No	27133379
	AA	Blood	81	13	30.8	90.1	qMSP	No	32373158
	AA	Stool	76	12	66.7	92.1	qMSP	No	32373158
*SFRP2*	CRC	Blood	37	47	72.3	89.2	MSP	No	28753106
		Stool	40	35	60.0	87.5	MSP	No	29142517
	Adenoma	Blood	37	37	83.8	89.2	MSP	No	28753106
	AA	Stool	40	36	27.8	87.5	MSP	No	29142517
*TFPI2*	CRC	Stool	53	61	93.4	94.3	qMSP	No	33958894
	Adenoma	Stool	53	16	81.3	94.3	qMSP	No	33958894
*Vimentin*	CRC	Blood	110	81	59	93	qMSP	No	19684580
		Stool	38	22	41	95	qMSP	No	19684580
	AA	Stool	38	20	45	95	qMSP	No	19684580
**Methylated gene panel**									
*NDRG4*, *BMP3*, mutation *KRAS*, hemoglobin	CRC	Stool	9167	65	92.3	86.6	Multitarget assay	Yes (2020)	24645800
	AA	Stool	9167	757	42.4	86.6			
*C9orf50, KCNQ5, CLIP4*	CRC	Blood	91	143	85	99	ddPCR	No	31727158
*MGMT, hMLH1, Vimentin*	CRC	Stool	37	60	75.0	86.5	MSP	No	19617759
	Adenoma	Stool	37	52	59.6	86.5		No	
*SFRP2, TFPI2, NDRG4, BMP3*	CRC	Stool	40	35	94.3	55.0	MSP	No	29142517
	AA	Stool	40	36	72.2	55.0		No	
*SDC2, TFPI2*	CRC	Stool	217	289	96.6	96.4	MSP	Yes (2022)	35004840
	Adenoma	Stool	217	190	80.0	95.7			
*SDC2, SFRP2*	CRC	Stool	1345	42	92.9	93.3	MSP	Yes (2022)	34933958
	AA	Stool	1345	302	35.1	93.3			
*SEPT9, SDC2, BCAT1*	CRC	Blood	60	104	82.7	96.9	MSP	Yes (2022)	34382948
*SDC2, NPY, FGF5, PDX1*	CRC	Stool	856	419	91.2	91.1	MSP	Yes (2023)	NA^&^
	AA	Stool	856	124	75.8	91.1			

^&^Retrieved from https://www.nmpa.gov.cn. AA, advanced adenomas; BS-HRM, bisulfite-specific high-resolution melting analysis; CRC, colorectal cancer; MSP, methylation-specific PCR; qMSP, quantitative methylation-specific PCR.

#### Stool-based DNA methylation biomarkers for screening and early detection of CRC

In addition to blood, stool is another promising sample source for CRC detection. Cancer cells released from tumor tissues accumulate in the stool, forming the basis for stool testing to identify tumor-specific hypermethylation changes and gene mutations. Numerous hypermethylated genes, including *BMP3, CDKN2A, FGF5, hMLH1, MGMT, NDRG4, NPY, PDX1, SDC2, SEPT9, SFRP2, TFPI2*, and *VIM*, have been analyzed in fecal DNA for CRC early detection^2,4^ (**Table 1**). Among these methylation-based CRC diagnostic biomarkers, methylated *VIM, BMP3, NDRG4*, and *SDC2* have demonstrated robustness for clinical use. Methylated *VIM* was the first stool-based methylation biomarker approved for CRC detection^8^; however, a meta-analysis involving 8 studies concluded unsatisfactory diagnostic performance of methylated *VIM*, with a sensitivity of 54.6% and a specificity of 88.5%^9^. Methylated *SDC2* was the first stool-based methylation assay for CRC detection approved by the NMPA in China. The sensitivity of methylated *SDC2* in fecal DNA for CRC was 83.8%, 42.1% for advanced adenomas, and 87.0% for early-stage CRC (stage I-II)^10^. Methylated *SDC2* appears to be the most accurate single gene among stool DNA methylation tests for detecting CRC based on a meta-analysis^9^, albeit large-sample clinical trials are needed for further validation.

#### Combined detection of multiple targets

Although single-gene methylation biomarkers have demonstrated promising specificity for CRC, the sensitivity is insufficient. Therefore, multigene combined testing, which has attracted much attention in recent years, may improve the sensitivity of CRC detection. Imperiale et al.^11^ proposed the use of FIT in addition to assessing *KRAS* mutations, aberrant *NDRG4*, and *BMP3* methylation for the early detection of CRC in stool samples. FIT demonstrated a 73.8% sensitivity and 94.9% specificity when used independently in CRC detection, and a 92.3% sensitivity and 86.6% specificity when combined with DNA testing^11^. Although the sensitivity of the multitarget stool DNA test did not vary significantly according to cancer stage or location within the colon, the sensitivity was relatively higher in distal advanced precancerous lesions than in proximal lesions (54.5% *vs.* 33.2%)^11^. This panel of multitarget stool DNA tests has been approved by the U.S. FDA and the NMPA in China for CRC diagnosis. In addition, the NMPA in China has approved several novel multigene methylation stool test kits for the detection of CRC, including *SDC2/TFPI2*, *SDC2/SFRP2*, *SEPT9/SDC2/BCAT1*, and *SDC2/NPY/FGF5/PDX1* (**Table 1**). The specificity of multigene combined testing is slightly lower than single-gene methylation testing, but the sensitivity is significantly better, which implies that multitarget combination testing is a promising future research domain.

#### Strengths and weaknesses between blood- and stool-based DNA methylation biomarkers for CRC detection

No head-to-head studies have compared the efficacy of these commercially available methylated gene detection kits in the same patient cohort. Based on studies with small sample sizes, methylated gene detection in stool samples did not demonstrate superiority over the detection of the same genes in plasma samples (**Table 1**). Of note, a blood sample can be obtained safely and objectively at any time, while a stool sample may not be collectible on demand. It is difficult to control feces quality and the characteristics of feces, such as loose or watery stools, may affect the test results. Moreover, fecal methylation testing cannot be used to monitor recurrence after surgical resection. Notably, the methylation biomarker detection rate in advanced adenomas was relatively low whether serum, plasma, or feces was analyzed. Although several methylation detection kits have been approved by the NMPA in China, it is important to note that the kits are a supplement to colonoscopy, not a replacement.

### Gastric cancer

Although early screening for gastric cancer *via* gastroscopy may improve overall survival^12^, the availability of reliable, simple, and non-invasive screening tests is more limited than for CRC. Several studies have recently been conducted to identify DNA methylation-based biomarkers in the plasma, serum, gastric juice, and fecal samples for gastric cancer diagnosis, albeit with varying specificity and sensitivity^13^. Early detection and *in vitro* diagnostics for gastric cancer have yet to reach clinics *en masse*.

#### Blood-based DNA methylation biomarkers for screening and early detection of gastric cancer

Several potential blood-based diagnostic methylation biomarkers have been identified for gastric cancer detection, including *C13orf18, DLEC1, FLNC, HODX10, MGMT, PCDH10, RNF180, RPRM, RPRML, RUNX3, SEPT9, SFRP2, SOX17, THBS1, UCHL1*, and *ZNF569*^13,14^ (**Table 2**). *RNF180* is one of the ring finger protein genes involved in the degradation of its substrates as an E3 ubiquitin ligase. Genes belonging to this family have been implicated in various biological processes, including cell growth, differentiation, and tumorigenesis^15^. Our previous study showed that the average methylation rate and methylated CpG sites within the *RNF180* promoter region in tissues increased with the severity of gastric mucosal lesions^16,17^. Therefore, methylated *RNF180* may serve as a candidate biomarker for gastric cancer. As mentioned earlier, methylated *SEPT9* has been identified as a non-invasive diagnostic biomarker for CRC; however, methylated *SEPT9* is not CRC-specific. Elevated levels of methylated *SEPT9* have been observed in various cancers, with 48%–56% of gastric cancer patients also testing positive for methylated *SEPT9*^18^. One study reported that the RS19 test is a new blood-based methylation assay for early gastric cancer detection that combines two methylated genes (*RNF180* and *SEPT9*) in a single reaction to improve the rate for early-stage gastric cancer and gastric dysplasia detection^14^. The RS19 test is an effective approach with good sensitivity (62.2%) and high specificity (84.8%) for detecting gastric cancer^14^. The plasma RS19 test has higher sensitivity than methylated *SEPT9* or *RNF180* alone in detecting gastric cancer and gastric dysplasia^14^. This study had the largest reported sample size, exceeding 1000 cases^14^. The RS19 test is the first epigenetic biomarker approved by the NMPA in China for detecting gastric cancer and is commercially available. Currently, the authors are conducting a multicenter community-based gastrointestinal cancer screening program using methylated *RNF180, SEPT9*, FIT, and *Helicobacter pylori* stool antigen (NCT05996458). In addition, another retrospective study presented a DNA methylation-based panel (*ELMO1, ZNF569*, and *C13orf18*) for distinguishing gastric cancer^19^. The study was limited by a relatively small sample size (36 patients with gastric cancer and 38 controls). It is anticipated that results from a larger study on screening, surveillance, or other intended-use populations will provide additional confirmation. Ongoing clinical trials are currently exploring the performance of novel blood DNA methylation-based panels for gastric cancer diagnosis (clinical trials.gov: NCT04511559, NCT04947995, NCT05224596, NCT05336058, NCT05347524, and NCT05668910; https://www.chictr.org.cn/: ChiCTR2300075157). Additional methylation kits for gastric cancer screening may become available for clinical use in the future.

**Table 2:** Overview of promising DNA methylation biomarkers used in the diagnosis of gastric cancer

Methylated sites	Sample type	Cohort size		Sensitivity (%)	Specificity (%)	Method	NMPA approval (date)	Reference PMID
		Controls	Cases					
**Single methylated gene**								
*DLEC1*	Blood	40	82	80.5	93	Q-MCP	No	26550574
*FLNC*	Blood	40	82	67.1	93	Q-MCP	No	26550574
*HODX10*	Blood	34	131	48.1	80	MSP	No	28529617
*PCDH10*	Blood	202	101	94.1	97.03	MSP	No	27330867
*RNF180*	Blood	527	650	46.2	87.3	MSP	No	37584087
*RPRM*	Blood	88	96	47	93	MSP	No	32431794
*RPRML*	Blood	25	25	56	88	MethyLight	No	33322837
*RUNX3*	Blood	34	131	42.7	79.2	MSP	No	28529617
*SDC2*	Stool	90	66	40.9	93.3	PCR	No	33765723
*SEPT9*	Blood	527	650	40.0	96.0	MSP	No	37584087
*SFRP2*	Blood	50	92	60.9	86	Q-PCR	No	32379490
*SOX17*	Blood	20	73	58.9	100	MSP	No	23403728
*THBS1*	Blood	40	82	63.4	94.2	Q-MCP	No	26550574
*TERT*	Stool	90	66	36.4	90	PCR	No	33765723
*RASSF2*	Stool	90	66	31.8	93.3	PCR	No	33765723
*SFRP2*	Stool	90	66	22.7	90	PCR	No	33765723
*UCHL1*	Blood	40	82	56.1	89.5	Q-MCP	No	26550574
*ZIC1*	Blood	34	131	69.5	69.2	MSP	No	28529617
**Methylated gene panel**								
*CABIN1, DOCK10, KCNQ5*	Blood	82	89	64	93	MCTA-Seq	No	34791072
*ELMO1, ZNF569, C13orf18*	Blood	38	36	86	95	MSP	No	29844130
*HODX10, RUNX3*	Blood	34	131	72.5	65	MSP	No	28529617
*MGMT, p15, hMLH1*	Blood	22	20	75	54	MSP	No	18837952
*RASSF2, SFRP2*	Stool	101	21	57.1	89.4	Hi-SA	No	19700653
*RNF180, SEPT9*	Blood	527	650	62.2	84.8	MSP	Yes (2020)	37584087
*RPRM, RUNX3*	Blood	88	96	82	89	MSP	No	32431794
*SDC2, TERT, hemoglobin*	Stool	90	66	66.7	78.9	PCR	No	33765723
*WIF1, SDC2, TFPI2, NDRG4*	Stool	107	35	67.5	97.81	ColoCaller	No	35419280
*ZIC1, RUNX3, HODX10*	Blood	34	131	91.6	50	MSP	No	28529617

MSP, methylation-specific PCR; MCTA-seq, methylated CpG tandem amplification and sequencing; Q-PCR, quantitative real-time PCR; q-MSP, quantitative methylation-specific PCR; Hi-SA, high-sensitivity assay for bisulfite DNA.

#### Stool-based DNA methylation biomarkers for screening and early detection of gastric cancer

Unlike CRC, only a few studies have investigated stool-based DNA methylation biomarkers for gastric cancer diagnosis (**Table 2**). Because the shedding of gastric tumor cells occurs in the upper gastrointestinal tract, tumor DNA passes through the intestines and is expelled from the body with feces after exposure to gastric acid, bile, and digestive enzymes. As a result, there is a minimal amount of tumor DNA available for testing in the stool. Existing studies have also shown that stool DNA methylation-based biomarkers do not exhibit good performance in detecting gastric cancer.

## Future developments and perspective

Although DNA methylation biomarkers outperform traditional markers, such as CEA, CA19-9, and CA125, in diagnosing early-stage gastric cancer and CRC, the overall sensitivity and specificity remain insufficient to fully meet the needs of cancer screening, especially for gastric cancer. Importantly, the impact of DNA methylation biomarker-based screening on reducing the incidence and mortality of gastrointestinal cancer remains unclear. Another potential limitation of DNA methylation biomarkers for routine cancer screening is the higher cost. To address these challenges and needs, several considerations are essential. First, specific combination algorithms are needed to better consolidate existing DNA methylation biomarkers and traditional tumor markers to improve the sensitivity of early cancer detection. Second, the genome has approximately 28 million CpG sites, which have enormous potential for mining. Therefore, it is necessary to mine and integrate novel methylation biomarkers as diagnostic targets using genome-wide profiling. Third, large randomized controlled trials are needed to verify whether DNA methylation marker-based cancer screening can reduce the incidence and mortality of gastrointestinal cancer. Concurrent health and economic evaluations during such trials are necessary to assess cost-effectiveness. Fourth, there is a stepwise accumulation of DNA methylation of tumor suppressor genes from precancers-to-cancers. Understanding whether patients without neoplastic lesions who test positive for DNA methylation biomarkers have a higher risk of developing cancer than the general population is also crucial. Therefore, quantitative detection and dynamic observation of DNA methylation levels may be helpful for these patients to determine whether or not the lesion is malignant.

Presently, all DNA methylation kits approved by the NMPA in China are used for the diagnosis of a single cancer type. The advantage of biomarkers for single cancer screening is the relatively clear identification of the corresponding target lesion in patients with positive detection. Moreover, the sensitivity and specificity of a single cancer methylation gene for cancer detection are high and the cost is relatively low, which warrants further development. However, a drawback is that for whole-body screening, multiple markers need testing with a substantial increase in costs. Therefore, pan-cancer DNA methylation biomarkers are more suitable for individuals undergoing whole-body cancer screening. PATHFINDER evaluated a pan-cancer early-detection blood test based on DNA methylation signatures^20^. The latest study supports the feasibility of this blood test for multicancer early detection^20^. Unfortunately, this pan-cancer screening technique overlooks 80% of early-stage tumors (stage I-II), indicating substantial room for improvement in sensitivity^20^. The U.S. FDA has approved this test as a groundbreaking advance, marking the commencement of a new era of global early cancer screening. Given the rapid advances in sequencing and analytical and computational technologies, DNA methylation biomarkers are emerging as a significant advance in optimizing cancer screening.
